# Evaluation of Race Pace Using Critical Swimming Speed During 10 km Open-Water Swimming Competition

**DOI:** 10.3390/jfmk10030302

**Published:** 2025-08-03

**Authors:** Yasunori Fujito, Tomomi Fujimoto, Reira Hara, Ryuhei Yoshida, Kazuo Funato

**Affiliations:** 1 Department of Sport and Health Sciences, Osaka Sangyo University, Osaka 574-0013, Japan; y-fujito@spo.osaka-sandai.ac.jp; 2 Department of Health and Sports, Niigata University of Health and Welfare, Niigata 950-3198, Japan; fujimoto@nuhw.ac.jp; 3 College of Sports Sciences, Nihon University, Tokyo 154-0002, Japan; hara.reira@nihon-u.ac.jp; 4 Kinoshita Group Co., Ltd., Tokyo 160-0023, Japan; yosida4075@yahoo.co.jp; 5 Graduate Schol of Sport System, Kokushikan University, Tokyo 206-8515, Japan

**Keywords:** long-distance swimming, endurance performance, physiological intensity, performance prediction

## Abstract

**Background**: Estimating race times for open-water swimming based on pool swimming times could be useful for talent identification and training optimisation. We aimed to compare the swimming speeds of the world’s top and other swimmers in the 2023 Aquatics Championship men’s 10 km OWS race. **Methods**: Sixty-five swimmers were divided into four groups: G1 (1st–10th positions), G2 (11st–30th positions), G3 (31st–47th positions), and G4 (48th–65th positions). Swimming speed, stroke frequency (SF), and stroke length (SL) for each lap (laps 1–6) were recorded. Critical speed (CS) was calculated from each participant’s personal best times in the 400, 800, and 1500 m freestyle events in the pool. Swimming speed against CS was calculated (%CS). **Results**: The top performance group (G1) maintained their swimming speed from beginning (lap 1, 1.53 m/s) to end (lap 6, 1.50 m/s), at 92.7 ± 1.9% of CS, characterised by longer SL (1.26 m) and lower SF (72.86 rpm). G3 and G4 were unable to maintain their swimming speed, which decreased from G3: 97.64 ± 1.62% and G4: 96.10 ± 1.96% of CS at lap 1 to G3: 88.39 ± 3.78% and G4: 85.13 ± 5.04% at lap 6. This reduction in swimming speed is consistent with the increased reliance on anaerobic metabolism reported in previous studies under similar conditions. **Conclusions**: Race pacing for maintaining speeds of 92%CS throughout the race could be an important resilient index in open-water swimming. %CS might be a useful index for estimating the athletic performance level in open-water swimming.

## 1. Introduction

Open-water swimming is performed in natural settings such as oceans, rivers, and lakes. The distance for open-water swimming in the Olympic Games is 10 km, with a finish time of approximately 2 h. Therefore, an optimal race pace suited to a swimmer’s ability is required to achieve the best performance, as open-water swimming involves currents, with no fixed lanes, unlike pool races. Studies on race pace for open-water swimming have demonstrated that top swimmers increase their swimming speed progressively from the beginning to the end of the race [1,2,3,4]. Specifically, towards the end of the race, top swimmers swim 1.5–3.0% faster than other swimmers [3,4]. Therefore, strategies for increasing swimming speed at the end of a race must be carefully considered.

Physiological indicators are commonly used to assess individual performance and optimal race pace. In previous studies, indoor 5 km race pacing occurred between 94–100% of the lactate threshold or critical speed (CS), which is close to race speed [5,6,7]; none have assessed race pacing during world-class open-water swimming races using a percentage of CS. Furthermore, determining exercise intensity relative to maximal oxygen uptake and the lactate work threshold, also used in previous studies, requires the aerobic capacity (i.e., maximum oxygen uptake and lactate threshold) of each swimmer to be measured before the race. Conversely, the aerobic capacity, CS, and finite work capacity beyond CS (D′: anaerobic work capacity) can be calculated using the relationship between the participant’s best time and distance; this is also used in swimming [8,9,10,11,12,13,14,15,16,17,18]. CS is the maximum intensity at which steady-state values of pulmonary gas exchange and muscle metabolic variables are stable [18]. Oxygen uptake is greater at exercise intensities of +2.5% of CS than during exercise at CS [19]. Therefore, it is expected that by identifying the race pace for open-water swimming using CS, an appropriate race pace for each swimmer can be determined.

Open-water swimmers often participate in pool swimming competitions. There is a difference in energy expenditure between open-water and pool swimming [20]. Furthermore, CS and performance in open-water swimming are strongly related [5,21]. In other words, as open-water swimming requires a different capacity than pool swimming, it was considered that estimating race times for open-water swimming based on pool swimming times could be useful for talent identification and training optimisation.

In this study, we aimed to assess the swimming speed of the world’s top swimmers and other swimmers in a 10 km open-water swimming race using their individual CSs. Furthermore, if OWS finish times can be estimated from personal bests in competitive swimming, this approach could be used to evaluate a swimmer’s abilities. Therefore, we aimed to clarify the feasibility of estimating the finish time for open-water swimming based on the relationship between personal best time and the distance used to calculate the CS. We hypothesised that the world’s top swimmers would maintain their swimming speeds close to their CS until the end of the race.

## 2. Materials and Methods

The race analysed in this study was the 10 km open-water sea event held during the 2023 World Swimming Championships in Fukuoka, on 15–16 July 2023. The course of the 10 km open-water swimming competition at the Fukuoka (2023) World Swimming Championships consisted of six laps of 1.66 km per lap. At the time of the race, the temperature was 29.6–30.0 °C, and the seawater temperature was 26 °C. Sixty-nine male swimmers participated in the study, of whom four failed to complete the swim. To compare the race pacing between swimmers of different competitive abilities, the swimmers were classified into four groups based on previous studies [21,22]. The groups were classified as G1, G2, G3, and G4 (1–10, 11–30, 31–47 and 48–65th, respectively). Publicly available data were obtained from the World Aquatics website (World Aquatics, https://www.worldaquatics.com). As these data are publicly available and accessible on the Internet, no formal ethics committee approval was necessary [21,22].

### 2.1. Data Analysis

Previous studies have reported that the season’s best times in the 400, 800, and 1500 m were attributed to male swimmers (competitions in 50 m pools, crawl) [16]. Therefore, we utilised the best official race times recorded between June 2022 and June 2023 for male swimmers to ensure recent and relevant performance data. CS and the curvature constant (D′) were subsequently determined by establishing a linear regression between swimming distance and completion time (Equation (1)).

y = ax + b(1)

where y is the distance swum, x is time, a is critical speed, and b is D′. In this regression model, the slope of the line represents CS, indicating the swimmer’s sustainable speed over longer distances, while the intercept corresponds to D′, reflecting the finite amount of work that can be performed above the critical speed before exhaustion occurs [14,15,16,17]. The model included swimmers with at least two distance and time results (e.g., 400 and 800, 800 and 1500 m, etc.) in pool competitions. Twelve swimmers with no pool competition results were excluded from the data, and the final number of participants was 53 swimmers. The predicted finish time for open-water swimming was calculated using linear regression equations for distance and time. Lap times and SF at the time of the race were checked on the World Swimming Championships website. This SF was obtained through accelerometers included in the timing bracelets (Swiss Timing, Swiss Timing Ltd., Corgémont, Switzerland) of each participant, which registered the number of swimming strokes. The swimming speed between each lap was calculated by dividing the lap time by the distance of the lap. The SL for each lap was calculated using Equation (2) [23,24]. The swimming speed relative to CS for each lap was calculated as a percentage of CS (%CS).

Stroke length = Swimming speed/Stroke frequency(2)

### 2.2. Statistical Analysis

Data are presented as mean ± standard deviation (SD). All data were examined for normal distribution using Q-Q plots. Mean differences in personal best time estimates, CS, D′, and race variables across the four performance groups (G1, G2, G3 and G4) were analysed using separate one-way analysis of variance (ANOVA). Post hoc comparisons were conducted using the Bonferroni method. To compare the pacing strategies between the groups, a two-way repeated-measures ANOVA (lap × groups) was employed. When statistical significance was detected, Bonferroni’s comparisons were performed. Statistical significance was set at *p* < 0.05 for all analyses. Pearson’s product–moment correlation coefficient was used to relate CS and D′ to race time. All statistical analyses were performed using the International Business Machines Statistical Package for the Social Sciences software for Windows (version 27.0; IBM Corp, Armonk, NY, USA).

## 3. Results

### 3.1. Parameter Estimates Based on Previous Race Performances

The top group (G1) showed significantly faster personal best times in the 400, 800, and 1500 m races, as well as higher CS values compared to the middle and lower groups (*p* < 0.05, Table 1). Times for the 400 m were lower in G1 and G2 than in G4, and similar trends were observed across groups for the 800 m, 1500 m, and CS. There were no significant differences in D′ between groups. The correlation coefficient between distance and time was r = 0.999.

### 3.2. Race Performance During 10 km Open-Water Swimming

The actual finish time of the 10 km races, swimming speed, and stroke variables are reported in Table 2. Finish time and mean swimming speed reduced in the order G1, G2, G3, and G4, with both measures being lower in that order (*p* < 0.05). SF and %CS were higher in G2 and G3 than in G4, and G1 did not differ from G4 (*p* < 0.05). SL was higher in G1 than in G3 (*p* < 0.05).

**Figure 1:**
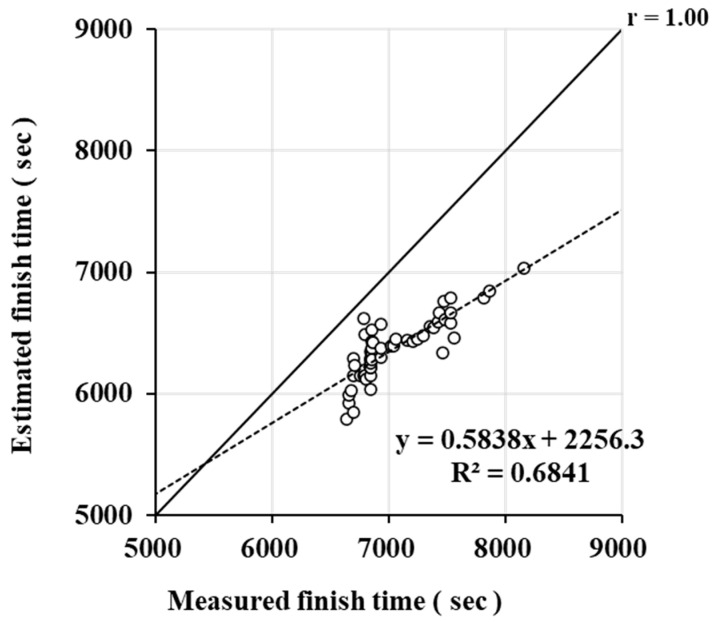
Comparison of measured and estimated finish time.

Figure 1 shows the relationship between the actual finish time of the 10 km open-water swimming and the estimated finish time from the model. The estimated finish time for OWS was calculated from the personal best–distance relationship, giving a value of 6373 ± 255 s. The actual measured finish time was 7051 ± 361 s. The actual measured finish time of open-water swimming was 9.5 ± 2.6% (678 ± 255 s) slower than the estimated finish time.

**Figure 2:**
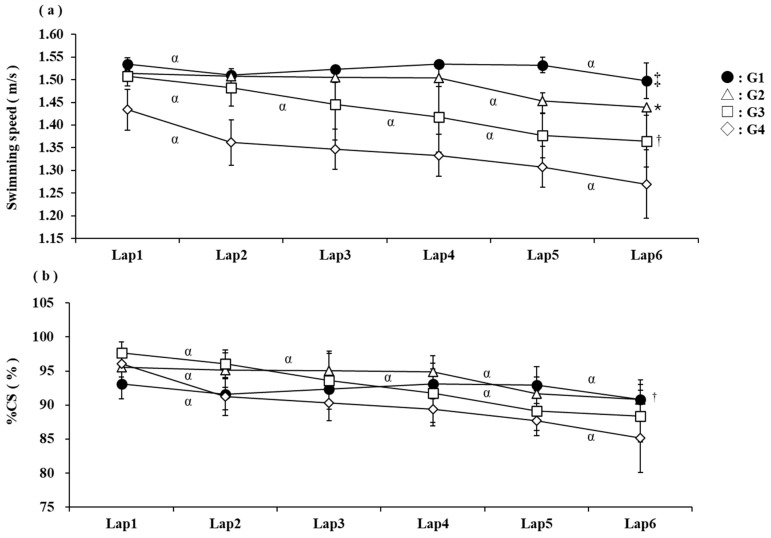
Comparison of swimming speed (**a**) and %CS (**b**) for each lap during the 10 km race. Differences with G3 and G4 are shown as * *p* < 0.05. Differences with G4 are shown as ^†^ *p* < 0.05. Differences with the other groups are shown as ^‡^ *p* < 0.05. Differences between previous splits are shown as ^α^ *p* < 0.05.

A comparison of swimming speeds for each lap of the 10 km open-water swimming race is reported in Figure 2a. The results showed that the main effects of the group factor, the lap factor, and the interaction were significant (F [3,49] = 96.53, *p* < 0.001; F [1,49] = 179.50, *p* < 0.001; F [3,49] = 18.47, *p* < 0.001, respectively). Multiple comparison tests showed that the swimming speeds of G3 and G4 decreased from the beginning to the end of the exercise. Conversely, the swimming speed of G2 was maintained up to lap 4 but decreased at lap 5. The swimming speed of G1 was also maintained up to lap 5 and decreased at lap 6. A comparison of %CS for each lap of the 10 km open-water swimming race is reported in Figure 2b. The results of the %CS showed that the main effect of the group factor was not significant, but the main effect and interaction of the wrap factor were significant (F [3,49] = 7.58, *p* < 0.001, F [1,49] = 178.47, *p* < 0.001, F [3,49] = 19.05, *p* < 0.001). The swimming speed was similar for %CS, but no differences existed between groups in G1, G2, and G3.

**Figure 3:**
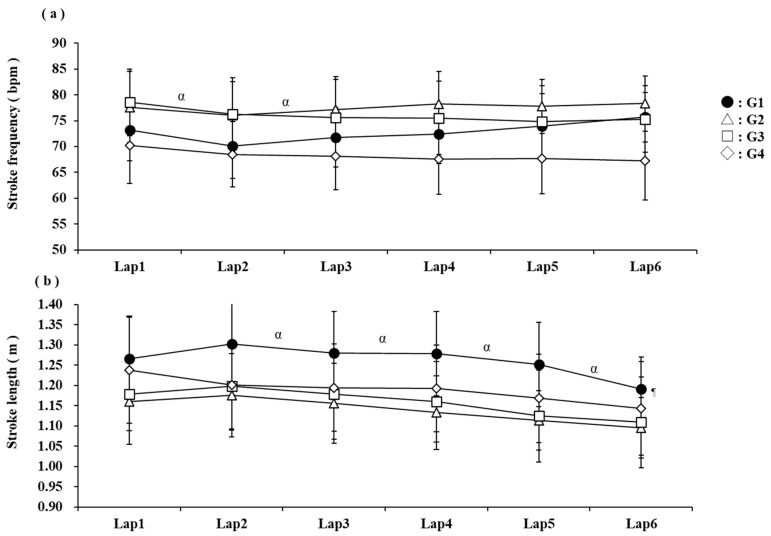
Comparison of SF (**a**) and SL (**b**) for each lap during the 10 km race. Differences with G3 are shown as ^¶^ *p* < 0.05. Differences between previous splits are shown as ^α^ *p* < 0.05.

A comparison of SF for each lap during the 10 km race is reported in Figure 3a. The results showed that the main effect of the group factor was not significant, and the main effect and interaction of the lap factor were significant (F [2,25] = 2.47, n.s., F [1,25] = 4.96, *p* < 0.05, F [2,25] = 1.53, n.s.). No significant difference existed in SF between the groups. Comparison between laps showed a decrease from one to two laps and an increase from two to three laps. A comparison of the SL for each lap during the 10 km open-water swimming race is reported in Figure 3b. The results showed that the main effect of the group factor, the main effect of the lap factor, and the interaction were significant (F [3,49] = 3.31, *p* < 0.05, F [1,49] = 72.32, *p* < 0.001, F [3,49] = 0.06, n.s.). The SL in G1 was higher than that in G3. No other differences existed between the groups. Comparing laps, a decreasing trend from lap 2 towards the end of the race was observed.

## 4. Discussion

In this study, we determined the swimming speed of the world’s top swimmers and other competitors in a 10 km open-water swimming race based on individual CSs. Furthermore, this study clarified the possibility of estimating race times for open-water swimming based on the relationship between personal bests and the distance used to calculate CS in competitive swimming. No differences in %CS existed between the groups, but the lower group tended to decrease from the initial phase of the race. In stroke variables, the top group had a longer SL and increased SF at the end of the race. Additionally, the estimated performance prediction value was overestimated by 9.5%.

### 4.1. Comparison of CS and D′ for Each Group

In this study, CS and D′ were calculated from personal bests in the pool at 400, 800, and 1500 m for all swimmers. The correlation coefficient obtained in this study (r = 0.999) was similar to that obtained in a previous study (r = 0.998–0.999) [14,15,16,17], confirming the validity of the model. In this study, the actual finish time was 9.5 ± 2.6% slower than the estimated finish time (Figure 1). This may be because the time was calculated from the relationship between distance and time for the estimation of time. In previous studies, the swimming efficiency is constant in the relationship between distance and time [15,16], whereas in open-water swimming, the swimming efficiency is not constant due to factors such as wind, waves, and lack of a defined course. Therefore, it is possible that these factors may cause prediction errors. However, since the effect of this prediction error is expected to be the same for all swimmers, performance predictions calculated from the best times of 400 m, 800 m, and 1500 m swimming races in the pool may be useful. In training, it is necessary to adjust training intensity and pacing strategies, considering the 9.5% difference in swimming speed between pool swimming and open-water swimming.

### 4.2. Comparison of Swimming Speed During the Race

Finish time and mean swimming speed decreased in the order G1, G2, G3, and G4 (Table 2), in line with previous research [1,2,3,4]. Conversely, the present study revealed that race pacing was assessed based on relative swimming speed as determined by CS. The %CS of G1 (92.31 ± 2.27%), G2 (93.84 ± 2.25%), G3 (92.72 ± 1.85%), and G4 (89.96 ± 1.83%) did not differ between the groups (Table 2). In a previous study, the %CS during the 5 km indoor event ranged from 94 to 100% [7], with the present study’s results being lower across all groups. This could be due to the indoor conditions in the previous study and the shorter competition distance.

Conversely, G3 and G4 were higher than G1 in %CS for each lap, and G3 and G4 were lower than G1 in lap 6 (Figure 2). As expected in a long event such as a 10 km race, the overall relative exercise intensity was in the moderate range. This is consistent with the fact that CS is a well-established indicator of moderate intensity exercise. However, detailed lap-by-lap analysis showed that G3 and G4 swimmers maintained speeds close to CS early in the race and decreased over time (Figure 2). This interpretation is consistent with the results of previous studies that have shown that excessively fast paces relative to the CS may cause early fatigue in long swims [9,21,25]. CS is used as the boundary between aerobic and anaerobic capacity [11,12,13,14,15,16,17,18] and is related to the ability to maintain speed [13]. Furthermore, maintenance of swimming speed in CS increases blood lactate levels [16]. Therefore, the race pace in open-water swimming may have been performed at an intensity approximately 10% lower than the CS to minimise the use of anaerobic capacity throughout the race, thus swimming with less fatigue. The use of drafting in the early stages of a race is important to make these races possible, as it has been reported to improve swimming efficiency [26]. Therefore, G3 and G4 may have been positioned for drafting early in the race and therefore may have been at speeds closer to CS. Therefore, it was suggested that to achieve high performance in the 10 km international OWS race, it is important to increase the swim speed around the CS and maintain around 92% CS from the beginning of the race to achieve high performance in the 10 km international open-water swimming race.

**Figure 4:**
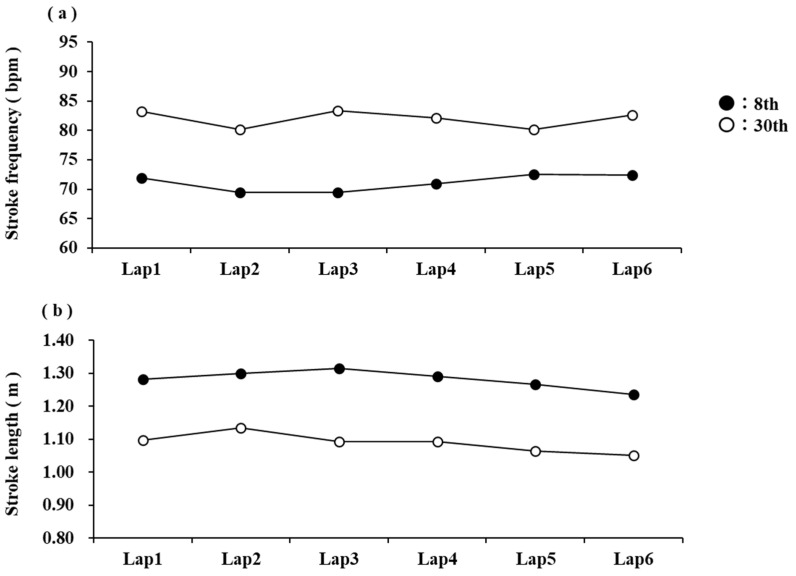
Comparison of SF (**a**) and SL (**b**) for each lap of swimmers with the same CS.

We also examined SF and SL to clarify the pacing strategy and swimming technique involved in achieving high performance in the 10 km open-water swimming race. Although no group differences existed in SF (Table 2), G1 showed an increase in SF in the final lap (Figure 3a). Conversely, G1’s SL was longer than the other groups throughout the race, but at the end of the race, SL was reduced to increase SF (Figure 3b). In long-duration competitions such as open-water swimming, the accumulation of fatigue makes it difficult to maintain SL [10], and swimmers tend to increase SF to maintain speed [1,2,3,4,22]. Race pacing was also assessed based on CS in this study. Among the participants, some had the same CS values but showed significant differences in rank, as illustrated in Figure 4. Figure 4 shows stroke variables for participants with the same CS. It can be observed that those with superior rankings had longer SL and lower SF throughout the race than those with inferior rankings. Studies examining the efficiency of different SF and SL at the same swimming speed have reported that swimmers with higher SL are more efficient than those with higher SF. These findings suggest that during open-water swimming races, longer SLs minimise energy expenditure and efficiently maintain a swim speed close to the CS, thereby increasing the number of swimming strokes in the end spurt.

### 4.3. Practical Applications

In this study, performance for open-water swimming was estimated based on competitive swimming times. As open-water swimmers often train in swimming pools, these findings can be used to adjust the intensity of pool training. Additionally, recent reports have described an increase in the number of dual swimmers who achieve high performance in both open-water swimming competitions and long-distance pool events. Estimating open-water swimming performance from personal best times in swimming may be a practical approach for evaluating potential participation in open-water swimming. Furthermore, our findings also revealed the importance of maintaining a high swimming speed while increasing the efficiency of swimming through long strokes during open-water swimming, and increasing SF during the final spurt, even when fatigued. These findings were useful from a physiological and technical perspective in terms of the kind of training necessary to improve performance in open-water swimming. These findings should be applied with caution because they are influenced by various environmental factors such as wind and waves. Future research should also consider the effects of environmental factors on race pace and physiological demands, as this will contribute to the development of race strategies in open-water swimming.

### 4.4. Limitations

In this study, the 2023 World Aquatics Championships held at Seaside Momochi Seaside Park in Fukuoka, Japan, served as the target race. Open-water swimming races are held in oceans and rivers, so wave and current conditions differ from event to event. Therefore, it is difficult to say that this study’s results correspond to the race pace of open-water swimming. In the future, it may be necessary to conduct research on the effects of weather and sea conditions by targeting more races. We did not measure blood lactate concentration, which is commonly used to quantify the anaerobic contribution in swimming studies [6]. This was due to the logistical and practical difficulties of collecting blood samples during open water races and our objective to analyse pacing in a real competition without the use of invasive procedures. Therefore, we used comparisons of CS and variations in swim speed and stroke-related indices as indirect indicators of physiological load and fatigue. Furthermore, this study was not compared with other open-water swimming studies. This is because of the difficulty of comparison due to the different weather and oceanographic conditions and distances involved [1,2,3,4,7]. Therefore, it should be noted that different race conditions may result in different race outcomes.

## 5. Conclusions

The top performance group (G1) maintained their swimming speed, from the beginning (lap 1) to the end (lap 6), at around 92.31 ± 2.27% CS, characterised by longer SL with lower SF. Contrarily, the lower performance groups (G3 and G4) were unable to maintain their relative swimming speed (%CS), decreasing from 98.7 ± 2.1% at lap 1 to 89.1 ± 4.4% at lap 6, which may contribute to significant intervention of anaerobic metabolism for energy production. We conclude that maintaining speeds of 92% of CS through the entire race could be an important resilient index in OWS. %CS, which was used in this study, might be a useful index for estimating the athletic performance levels in OWS.

## Figures and Tables

**Table 1:** Comparison of the personal best of pool swimming, CS, and D′ for each group.

			G1	G2	G3	G4	F	*p*
400 m time	s	mean	231 ± 4 *	238 ± 7 ^†^	242 ± 8	250 ± 10	F (3,44) = 11.52	<0.001
		CI	(228–233)	(235–242)	(238–246)	(244–257)		
800 m time	s	mean	472 ± 9 ^α^	489 ± 11 ^†^	496 ± 14 ^†^	514 ± 17	F (3,46) = 17.57	<0.001
		CI	(466–478)	(484–494)	(489–503)	(503–525)		
1500 m time	s	mean	898 ± 21 ^α^	930 ± 22 *	953 ± 16 ^†^	984 ± 30	F (3,47) = 27.29	<0.001
		CI	(885–911)	(919–941)	(944–961)	(964–1003)		
Critical speed	m/s	mean	1.65 ± 0.05 ^α^	1.59 ± 0.04 *	1.54 ± 0.03 ^†^	1.49 ± 0.04	F (3,49) = 32.81	<0.001
		CI	(1.62–1.68)	(1.57–1.60)	(1.53–1.56)	(1.46–1.52)		
D′	m	mean	19.83 ± 9.12	24.10 ± 8.50	30.80 ± 14.61	29.82 ± 7.87	F (3,49) = 2.89	0.05
		CI	(14.18–25.48)	(19.94–28.26)	(22.86–38.26)	(24.68–34.96)		

Differences with G3 and G4 are shown as * *p* < 0.05. Differences with G4 are shown as ^†^ *p* < 0.05. Differences with the other groups are shown as ^α^ *p* < 0.05.

**Table 2:** Comparison of finish time, swimming speed, and stroke variables for each group.

			G1	G2	G3	G4	F	*p*
Finish Time	s	mean	6699 ± 45 *	6834 ± 25 *	7153 ± 198 ^†^	7643 ± 230	F (3,49) = 89.09	<0.001
		CI	(6667–6731)	(6822–6847)	(7044–7263)	(7479–7808)		
Swimming speed	m/s	mean	1.52 ± 0.01 ^α^	1.49 ± 0.01 *	1.43 ± 0.04 ^†^	1.34 ± 0.04	F (3,49) = 96.32	<0.001
		CI	(1.51–1.53)	(1.48–1.49)	(1.41–1.45)	(1.31–1.37)		
Stroke frequency	rpm	mean	72.86 ± 5.66	77.52 ± 5.68 ^†^	75.59 ± 6.60 ^†^	68.24 ± 6.71	F (3,49) = 5.29	<0.001
		CI	(68.81–76.90)	(74.70–80.35)	(71.94–94.24)	(63.44–73.04)		
Stroke length	m	mean	1.26 ± 0.10 ^¶^	1.16 ± 0.09	1.14 ± 0.11	1.19 ± 0.11	F (3,49) = 3.30	0.05
		CI	(1.19–1.33)	(1.12–1.20)	(1.08–1.20)	(1.11–1.27)		
%Critical speed	%	mean	92.31 ± 2.27	93.84 ± 2.25 ^†^	92.72 ± 1.85 ^†^	89.96 ± 1.83	F (3,49) = 7.58	<0.001
		CI	(90.68–93.93)	(91.96–93.75)	(91.69–93.75)	(91.63–93.17)		

Differences with G3 and G4 are shown as * *p* < 0.05. Differences with G4 are shown as ^†^ *p* < 0.05. Differences with G3 are shown as ^¶^ *p* < 0.05. Differences with the other groups are shown as ^α^ *p* < 0.05. CI, confidence interval.

## Data Availability

The data used in this study are publicly accessible from the official website of World Aquatics (https://www.worldaquatics.com).

## Abbreviations

The following abbreviations are used in this manuscript:

SF	Stroke frequency
SL	Stroke length
CS	Critical speed
ANOVA	Analysis of variance
